# Studying GGDEF Domain in the Act: Minimize Conformational Frustration to Prevent Artefacts

**DOI:** 10.3390/life11010031

**Published:** 2021-01-06

**Authors:** Federico Mantoni, Chiara Scribani Rossi, Alessandro Paiardini, Adele Di Matteo, Loredana Cappellacci, Riccardo Petrelli, Massimo Ricciutelli, Alessio Paone, Francesca Cutruzzolà, Giorgio Giardina, Serena Rinaldo

**Affiliations:** 1 Department of Biochemical Sciences “A. Rossi Fanelli”, Sapienza University of Rome, Piazzale Aldo Moro, 5, 00185 Rome, Italy; federico.mantoni@gmail.com (F.M.); chiara.scribanirossi@uniroma1.it (C.S.R.); alessandro.paiardini@uniroma1.it (A.P.); alessio.paone@uniroma1.it (A.P.); francesca.cutruzzola@uniroma1.it (F.C.); 2 Laboratory Affiliated to Istituto Pasteur Italia—Fondazione Cenci Bolognetti, 00185 Rome, Italy; 3 Institute of Molecular Biology and Pathology, CNR, Piazzale Aldo Moro, 5, 00185 Rome, Italy; adele.dimatteo@cnr.it; 4 Medicinal Chemistry Unit, School of Pharmacy, University of Camerino, Via S. Agostino 1, 62032 Camerino, Italy; loredana.cappellacci@unicam.it (L.C.); riccardo.petrelli@unicam.it (R.P.); massimo.ricciutelli@unicam.it (M.R.)

**Keywords:** enzyimatic assay, GGDEF, c-di-GMP, allostery, protein engineering

## Abstract

GGDEF-containing proteins respond to different environmental cues to finely modulate cyclic diguanylate (c-di-GMP) levels in time and space, making the allosteric control a distinctive trait of the corresponding proteins. The diguanylate cyclase mechanism is emblematic of this control: two GGDEF domains, each binding one GTP molecule, must dimerize to enter catalysis and yield c-di-GMP. The need for dimerization makes the GGDEF domain an ideal conformational switch in multidomain proteins. A re-evaluation of the kinetic profile of previously characterized GGDEF domains indicated that they are also able to convert GTP to GMP: this unexpected reactivity occurs when conformational issues hamper the cyclase activity. These results create new questions regarding the characterization and engineering of these proteins for in solution or structural studies.

## 1. Introduction

**Scheme 1:**
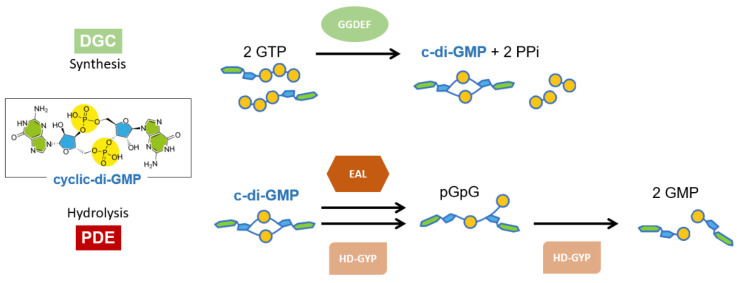
Synthesis and degradation of cyclic diguanylate (c-di-GMP).

Bacterial biofilm depends on the modulation of cyclic diguanylate (c-di-GMP) levels in response to environmental cues. This second messenger is also involved in bacterial signal transduction, regulation, virulence and antibiotic resistance. It is synthesized by diguanylate cyclases (DGCs) harboring the GGDEF domain, starting from two GTP molecules. Conversely, it is degraded by dedicated phosphodiesterases (PDEs), belonging to two families namely EAL and HD-GYP, to 5′-phosphoguanylyl-(3′-5′)-guanosine (pGpG). Moreover, the HD-GYP family is also able to carry out a second hydrolytic cleavage to produce GMP through the so-called PDE-B activity, via the c-di-GMP → pGpG → GMP pathway (Scheme 1) [1]. GGDEF, EAL and HD-GYP are named according to the sequence signature in the active site. These enzymatic domains are very often fused to a plethora of regulatory domains (e.g., REC; GAF; PAS; PAC; HAMP), and a large number of homologous but non-redundant multidomain enzymes per species exist, whose response to environmental stimuli is either direct or mediated by protein-protein interaction with other sensors [1]. Many receptors able to sense the cellular levels of the dinucleotide have been identified, including transcription factors, PilZ domains, degenerate DGCs or PDEs, and also riboswitches [2].

The variety of domain architectures in c-di-GMP metabolizing enzymes and the heterogeneity of its receptors, allow bacteria to react promptly to many different environmental conditions by modulating their c-di-GMP based adaptation strategy. As a consequence, it has been very hard to draw a unique mechanism for the c-di-GMP-mediated regulation. The only general rule that has emerged to date, is that the c-di-GMP dependent modulation of protein function occurs mainly via restriction of the possible conformations accessible to these multi-domain or multi-subunit proteins [3,4].

**Scheme 2:**
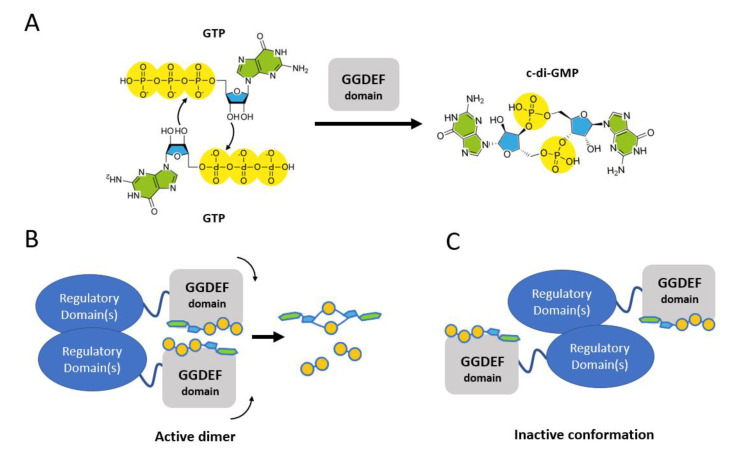
(**A**) In order to yield c-di-GMP, two GTP molecules must come in close proximity. Since each GGEDF domain binds only one GTP molecule, for the condensation reaction to occur two GGDEF domain must dimerize as shown in panel (**B**). The GGDEF containing enzymes respond to environmental stimuli by switching from an active conformation (**B**) to an inactive one in which the two GGDEF domain are not able to interact (panel (**C**)).

The GGDEF domain of DGCs is emblematic of this control; since only one GTP molecule can bind in the active site, a dimerization step is mandatory for the cyclization reaction to occur and yield c-di-GMP. Consequently, multidomain DGCs usually regulate their activity by allowing the GGDEF domains to dimerize (active dimer) or forcing them apart (inactive conformations), functioning as conformational switches (Scheme 2).

On the other hand, although in principle the hydrolysis of c-di-GMP could be performed by a single EAL domain, EAL containing proteins have been also shown to function as dimers and, as the GGDEF domains, to function as conformational switches [5,6,7,8]. Finally, the GGDEF domain can also be found fused at the N-terminus of an EAL domain, in the so-called hybrid proteins which potentially could display opposite catalytic activities. However, in these intriguing enzymes, usually only one of the two domains is active, with the inactive domain serving as allosteric regulator. In this scenario, it has been often reported that the GGDEF domain may control the catalytic properties of the downstream EAL domain of hybrid proteins [5,8,9,10]. The GGDEF-containing proteins represent a hot target to interfere with biofilm formation [11], including the rationale approach targeting the active or the allosteric sites [12,13,14,15,16,17]. However, to improve the target specificity, novel functional and structural data are required. From a biochemical point of view, working with such multi-domain proteins in vitro poses several issues which need to be taken into careful consideration in order to achieve a reliable characterization. Most importantly, given the many regulatory and/or transmembrane domains associated to DGCs, PDEs or hybrid proteins, these are often poorly soluble and shorter constructs that must be expressed to study them in vitro. Moreover, truncation of one or more domains may result in a modification of the allosteric regulation, whose characterization is the main goal of the study. Consequently, even when the design of the constructs is cautiously done, misleading results may be obtained.

Here we report a re-evaluation of the kinetic profile of selected hybrid proteins as truncated catalytic constructs, showing that the GGDEF domain can slowly convert GTP to GMP as a side reaction. We also show that different GGDEF-containing proteins display this unexpected reactivity, and that this side reaction occurs when the physiological cyclase activity does not take place due to conformational or dimerization issues. We found that when using GTP as a substrate the GGDEF domain may catalyze opposite reactions (condensation or hydrolysis) depending on the presence of upstream domain(s) or on the assay conditions. This variability may bias the kinetic characterization of these proteins and raises some concerns on the design of soluble constructs of GGDEF-containing proteins. This work aims at highlighting these peculiar properties of the GGDEF domain and to propose some useful guidelines to minimize the structural and kinetics bias due to a possible disruption of the GGDEF conformational constraints in engineered constructs.

## 2. Materials and Methods

### 2.1. Protein Expression and Purification

RmcA-DUAL construct and site-directed mutant was obtained and purified as previously published [5]. Synthetic genes PA0861 (Rbda, residues 373–818) and PA4601 (MorA, residues 970–1409) from *P. aeruginosa* harboring only the GGDEF and the C-terminal EAL domains were purchased from Life Technologies and subcloned in Pet28a vectors with an N-terminal His-tag between NdeI and XhoI/HindIII. YfinN GGDEF and PleD were obtained as reported in [18,19], respectively. Purification of each construct was done as reported below, and the corresponding buffers for each construct were reported in Table S1. All constructs were overexpressed in the *Escherichia coli* BL21 (DE3) strain. Freshly transformed colonies were grown at 37 °C in Luria-Bertani (LB) liquid medium supplemented with 30 μg/mL Kanamycin (Merck KGaA-Germany). At OD600 ~0.8, protein expression was induced by adding 0.5 mM IPTG (isopropyl Β-d-thiogalactoside; Merck KGaA-Germany) and the temperature was reduced to 20 °C. Cells were harvested by centrifugation after 20 h and stored at −20 °C. All bacterial pellets were suspended in the lysis buffer and then they were lysed by sonication. The supernatant was separated on a Ni^2+^-IMAC HisTrap column (GE Healthcare, Chicago, IL, USA) equilibrated in Buffer A (Table S1), specific for each protein. Elution was carried out by increasing the imidazole concentration, with the proteins eluting at 300 mM imidazole. Fractions containing pure protein were pooled, imidazole was removed with PD-10 desalting columns (GE Healthcare, Chicago, IL, USA) and concentrated with Ultracel Amicon^®^ concentrators (Merck KGaA-Germany). Monodisperse protein was obtained by FPLC, according to their molecular weights, and eluted with buffer A using an FPLC apparatus (AKTA system, GE Healthcare, Chicago, IL, USA) (Table S1). For PleD, multiple FPLC runs were done on diluted protein to allow c-di-GMP to dissociate on the column and then the collected dimer peak was concentrated. Purified proteins were flash-frozen in liquid nitrogen and stored at −20 °C and the protein content was evaluated with BCA assay (Merck KGaA-Germany) and spectroscopically. When affordable the Molar Extinction coefficient (ε) was calculated by BCA.

### 2.2. Kinetic Assay GTPase Activity

The (α-β)-GTPase activity (hereinafter GTPase) of different RmcA derived constructs as well as of PleD, YfiN, RbdA and MorA was analyzed using Reverse Phase HPLC (RP-HPLC) as reported in [19]. Small variations in peak migration are ascribed to the difference in the room temperature during RP-HPLC runs; purified standards were run routinely to set the peak (data not shown). RmcA derived constructs and YfiN (10, 8 or 5 μM, as indicated) in 20 mM TrisHCl pH 8.0, 100 mM NaCl and 2.5 mM MnCl_2_ were incubated with 100 μM GTP and the reaction mixture was kept at 25 °C for different time intervals. No activity was observed when Zn^2+^ or Ca^2+^ were used as divalent cations (data not shown). The rates of hydrolase activity were also assayed in the presence of 30 or 60 μM c-di-GMP, if indicated. To test the putative GTPase activity of PleD, 5 μM of enzyme in 20 mM TrisHCl pH 8, 100 mM NaCl and 10 mM MgCl_2_ was incubated for 10 min at room temperature. To populate the active DGC form, if indicated, the enzyme was incubated at room temperature for 30 min with 10 mM NaF and 1 mM BeCl_2_. All the reactions, which were carried out with different enzymes, were stopped as described in [5]. All the experiments were done at least in triplicate; a sample experiment is reported in figures. Rates have been obtained as the linear fit of the initial time course and are the mean value ± SD. As a control, the reaction with the sole GTP was also carried out to monitor eventual auto hydrolysis of GTP; no degradation (and therefore GMP accumulation) was observed (data not shown).

### 2.3. Optimization of GMP Separation by RP-HPLC for Mass Spectrometry

To assess the identity of the GTPase activity product, we optimized the RP-HPLC separation. We separated the unknown species using a 150 × 4.6 mm reverse phase column (Prevail C8, Grace Davison Discovery Science, particle size of 5 μm). Due to the incompatibility of 100 mM phosphate buffer with MS analysis, catalytic products were eluted with 0.1% Formic Acid/Methanol (98/2, *v*/*v*, 0.3 mL/min) a mobile phase, setting the UV detector at 254 nm. The product eluted was lyophilized and Mass Spectrometry analysis was done. HPLC/DAD/ESI-TRAP studies were performed using an Agilent 1100 series and an Ion Trap LC/MSD Trap SL G2445D from Agilent Technologies (Santa Clara, CA, USA) equipped with an ESI source operating in negative ionization mode.

### 2.4. Crystallography

YfiN_GGDEF_ crystals were obtained by vapor diffusion methods (sitting drop) by mixing equal volumes of 2.5 mg/mL protein solution (buffer: 250 mM NaCl 10 mM Tris pH 8.0, 2% glycerol, 5 mM MgCl_2_) with precipitant solution (0.2 M ammonium acetate, 0.1 M Tri-sodium Citrate pH 5.5, 30% *w*/*v* PEG 4K). Before flash freezing in liquid nitrogen, crystals were soaked in a solution of mother liquor containing 20% glycerol as cryoprotectant. A 360° data-set (oscillation of 0.1°) was collected at the BESSY synchrotron on ID-14.1 beamline, at 0.918 wavelength. The best crystal diffracted to 2.8 Å resolution. Data were integrated using XDS [20]. Scaling, conversion of intensities to amplitudes, systematic absence analysis and free flag (10%) assignment was performed using Aimless [21] within the CCP4 [22] software suite. Initial phases were obtained by molecular replacement using the PDB entry 4IOB [18] as search model in MOLREP [23] Cycles of model building and refinement were done using COOT [24] and Refmac-v5.8 [25] respectively. Data collection and refinement statistics are shown in Table 1. Coordinates and structure factors were deposited in the protein data bank with accession code: 7A7E.

## 3. Results

### 3.1. The GGDEF Domain of RmcA Is Able to Hydrolyze GTP

**Figure 1:**
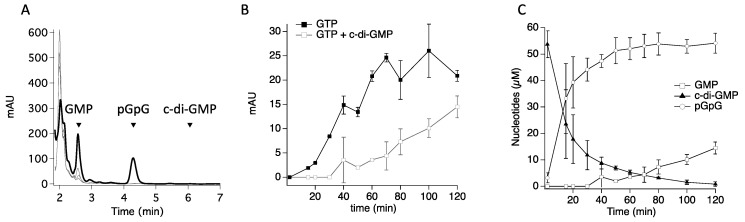
GMP production by DUAL. (**A**) Chromatograms showing the nucleotide content of the reaction of 5 µM DUAL with 100 µM GTP: pGpG accumulates (black line) only after 18 h of incubation (k_cat_ 0.035 min^−1^), thus suggesting that DUAL is unlikely to be a DGC under physiological conditions. Incubation times lower than two hours yields only a species eluting at 2.7 min, which is GMP (grey lines, see Figure S1 for details). (**B**) Time-course of GMP production performed with 8 μM DUAL, in the presence of 60 µM c-di-GMP or in its absence (as indicated in the legend), under experimental conditions optimized for GTPase activity. (**C**) Nucleotide content of the reaction described in (**B**) in the presence of c-di-GMP, as a function of time. Data are the mean of three experiments ±SD. Peaks below the GMP one are due to GTP and buffer components.

We recently characterized the catalytic moiety of RmcA from the *P. aeruginosa* strain PAO1, a transmembrane GGDEF-EAL containing phosphodiesterase able to recognize L-Arginine in the periplasmic space [26] and to degrade c-di-GMP [5]. We demonstrated that the GGDEF domain binds GTP and allosterically controls the phosphodiesterase activity by allowing the EAL domain to bind to c-di-GMP and dimerize [5]. Given that the GGDEF domain can bind GTP and metals as a genuine DGC, we decided to re-evaluate possible activities of GGDEF occurring in parallel to the characterized PDE one of the GGDEF-EAL (hereinafter DUAL) construct. Therefore, we analyzed the activity of DUAL in the presence of the sole GTP (excess, 100 µM) at protein concentration higher than that previously used (5–8 μM DUAL rather than 1 μM). Under these experimental conditions, where pseudo-first order is still guaranteed, two different species accumulate: an unexpected early one (Figure 1A), found to be GMP by mass spectrometry (Supplementary Figure S1) and a late one (appreciable after many hours) corresponding to pGpG (Figure 1A and Supplementary Figure S1, panel D). The production of pGpG likely belongs to a residual DGC activity of the GGDEF domain, which slowly forms c-di-GMP, rapidly converted into pGpG by the EAL domain.

Interestingly, when GTP and c-di-GMP are both present in the reaction mixture, we observed that GMP accumulates after a lag-phase of ~35 min (Figure 1B), contrary to the reaction with the sole GTP. During this lag-phase, c-di-GMP is actively consumed by the PDE domain (Figure 1C) and only when residual c-di-GMP reaches ~8 µM, GMP accumulation is observed (Figure 1C).

### 3.2. (α-β)-GTPase Activity on Other Hybrid Proteins

**Figure 2:**
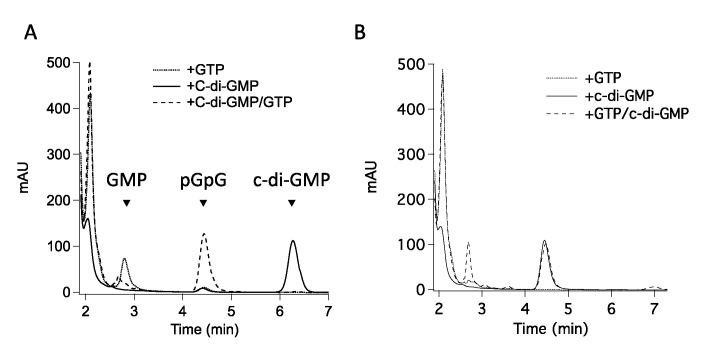
Kinetic properties of other hybrid proteins. (**A**) RP-HPLC chromatograms of 1 h of reaction of 10 µM MorA with 100 µM GTP or 30 µM c-di-GMP or with both nucleotides. (**B**) The same experiments reported in (**A**) were run with 10 µM RbdA. A sample experiment of a triplicate is shown; the peaks below the GMP one are due to GTP and buffer components.

To investigate whether GTP hydrolysis is a general feature of the GGDEF-EAL DUAL moiety, we qualitatively analyze the reactivity of the motility regulator protein MorA from *P. aeruginosa*, whose kinetic characterization has been previously carried out [27]. Briefly, Phippen and coworkers found that MorA is able to convert GTP to c-di-GMP through its GGDEF domain and to hydrolyze c-di-GMP to pGpG by the EAL domain, in the presence of the sole GTP or with both GTP and c-di-GMP; on the other hand, MorA phosphodiesterase activity is almost absent in the absence of GTP. The same profile has been observed in this study and no GMP accumulation occurs (Supplementary Figure S2A–C). Nevertheless, experiments carried out under the conditions optimized for RmcA, i.e., ~4 folds lower protein concentration and pseudo-first order conditions, only confirm that MorA is a GTP-dependent PDE; no DGC activity is detectable when GTP is the sole substrate (Figure 2A) while GMP accumulates when GTP is present. Therefore, reactivity of MorA with GTP depends on the experimental conditions.

We then analyzed the reactivity of another well-characterized hybrid protein: the positive regulator of biofilm dispersal RbdA from *P. aeruginosa* [8]. Under our experimental conditions, the DUAL portion of RbdA displays PDE activity and lacks the DGC one; moreover, in the presence of both GTP and c-di-GMP, GMP also accumulates (Figure 2B). The reactivity of this construct is different from that observed by Liu and coworkers, who found that the protein is both a DGC and a GTP-enhanced PDE, with no GMP accumulation [8]. Nevertheless, they worked on a RbdA construct harboring PAS-PAC-GGDEF-EAL domain, which is different from ours and harbors only the DUAL moiety; the PAS-PAC portion upstream the GGDEF domain likely allows GGDEF to productively dimerize and to populate a catalytically competent DGC able to enter catalysis.

### 3.3. GMP Production Is Not Due to an Unexpected PDE-B Activity of the EAL Domain

**Figure 3:**
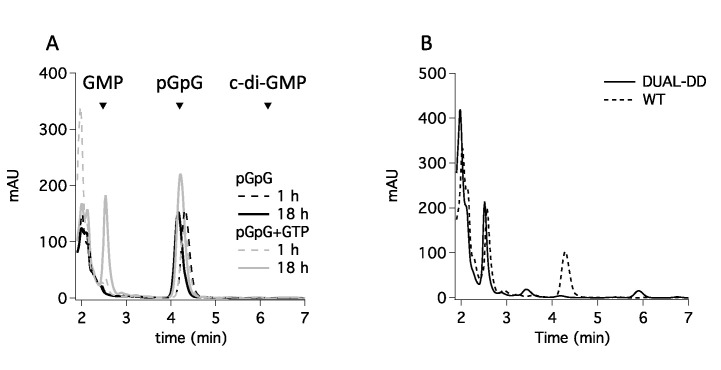
GMP does not belong to a PDE-B activity. (**A**) Chromatograms showing the nucleotide content of the reaction of DUAL (5 µM) with 30 µM pGpG with or without 100 µM GTP (grey and black traces, respectively) at 1 h or 18 h (dashed and continuous lines, respectively). GMP accumulates only in the presence of GTP. (**B**) Chromatograms showing the nucleotide content of the reaction of 5 µM DUAL or DUAL-DD (dotted or continuous line, respectively), with 100 µM GTP after 18 h of incubation. GMP accumulates in both samples while pGpG is produced only in the DUAL sample. A sample experiment of a duplicate is shown; peaks below the GMP one are due to GTP and buffer components.

Given that GMP production is a common trait of the GGDEF-EAL tandem, a series of experiments were done to exclude a side (and unusual) PDE-B activity. As mentioned above, pGpG may be further hydrolyzed into 2 molecules of GMP (the so-called PDE-B activity), as observed in the HD-GYP subtype of PDEs [27]. To probe or exclude this, we investigated the reactivity of RmcA DUAL with the sole pGpG. Kinetics data indicate that no pGpG depletion occurs even in the presence of GTP, while GMP accumulation is observed only when GTP is present (Figure 3A). These findings are consistent with what previously reported on the isolated RmcA EAL domain, which, although efficient as PDE, is not able to populate GMP from c-di-GMP or pGpG [5].

Moreover, we tested the reactivity of the double mutant of DUAL in the EAL domain D1136N/D1137N (hereinafter DUAL-DD), where the replacement of the two aspartic acid residues hampers the holo active site construction and finally PDE turnover [5]. As shown in Figure 3B, GMP still accumulates in the presence of GTP. To a lower extent also c-di-GMP is formed, belonging to a spurious DGC; c-di-GMP is not further hydrolyzed into pGpG since the PDE activity is functionally abolished by mutation. In the wildtype protein, on the contrary, the little amount of c-di-GMP, due to the spurious DGC, is rapidly hydrolyzed into pGpG, as commented above. Taken together, all these data indicate that conversion of GTP into GMP does not belong to a PDE-B activity.

### 3.4. GGDEF Incompetent Dimerization Promotes GMP Production

**Figure 4:**
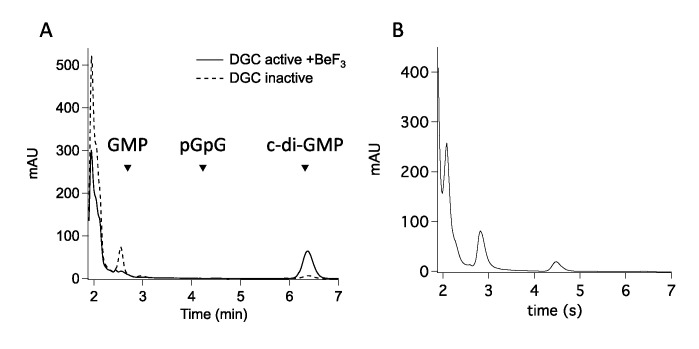
The GGDEF domain of other DGCs is able to produce GMP. (**A**) RP-HPLC chromatograms of PleD DGC reaction. Catalytic reaction was carried out either using PleD enzyme activated as DGC by BeF3 (continuous lines) or with the inactive form of the enzyme, as purified (dotted lines). (**B**) RP-HPLC chromatograms of YfiN DGC reaction. The little amount of pGpG which accumulates after 2 h could be ascribed to the α-ß hydrolysis of the DGC intermediate pppGpG, thus further confirming the aberrant behavior of a truncated GGDEF. A sample experiment of a triplicate is shown; peaks below the GMP one are due to GTP and buffer components.

If this background reactivity is a general feature of the GGDEF domains, it could bias kinetic studies describing GGDEF-containing proteins; therefore, we probed the reactivity of GGDEF domains with GTP in protein with domain architectures lacking the DUAL motif. We analyzed the reactivity of PleD from *C. crescentus*, since it represents the reference system for studying DGCs: this protein populates a stable inhibited state, which is released by phosphorylation to allow the GGDEF moieties to interface and enter catalysis upon GTP binding [28]. As shown in Figure 4A, reaction of inhibited PleD (“as purified”) with GTP yields GMP, while c-di-GMP synthesis was negligible. As expected, the activated PleD (treated with beryllium fluoride to mimic phosphorylation) recovers the DGC activity yielding c-di-GMP, while no GMP is observed (Figure 4A). Therefore, GMP accumulates from GTP when the productive dimerization of the GGDEF domains, necessary for the DGC activity of PleD [29], is conformationally hampered.

Since the dimer formation is a pre-requisite to enter DGC catalysis, we investigated the possible GMP production of the isolated GGDEF of YfiN from *P. aeruginosa*, an inactive truncated version, previously shown to be a monomer able to bind GTP with high affinity [18]. According to our hypothesis, the kinetic data clearly shows that the GGDEF domain of YfiN displayed the side activity accumulating GMP, while c-di-GMP production absent (Figure 4B).

### 3.5. Structure of Inactive YfiN_GGDEF_

**Figure 5:**
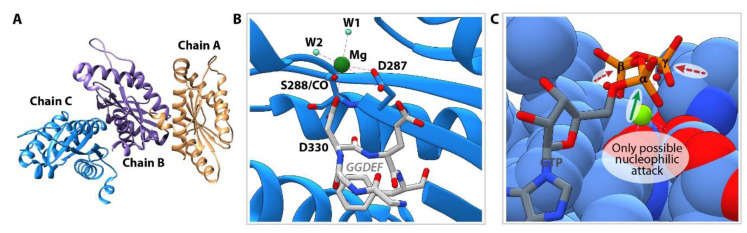
Structure of a monomeric GGDEF (YfiN_GGDEF_). (**A**) Content of the asymmetric unit showing 3 molecules. Each chain consists of a single GGDEF domain. (**B**) Close-up view of the GTP binding site (chain C). The residues belonging to the GGDEF signature are colored in white. The magnesium ion (dark green) is coordinated by the main-chain carboxyl group of S288, the carbonyl groups of D287 and D330 (GGDEF) and two water molecules. (**C**) Structural superposition of GTP (grey) and calcium (light green) as observed in complex with chain B of RmcA (PDB id: 5m3c [5])—and YfiN GGDEF (blue). The structure of YfiN GGDEF is shown as spheres to highlight the steric hindrance surrounding the GTP molecule. The direction of a possible nucleophilic attack at the α-phosphate is indicated with a black arrow, the other phosphate groups appear much less accessible due to the presence of the surrounding protein residues.

The versatile reactivity of the GGDEF domain seems to be related to a conformational issue rather than to an altered active site (as compared to the catalytically competent site required for DGC reaction): this may explain the alternative reactivity of the same protein towards GTP, depending on the allosteric status of the protein. This hypothesis has been confirmed by solving the 3D structure of the YfiN_GGDEF_ protein in complex with Mg^2+^ by X-ray crystallography at 2.8 Å resolution. The crystal contained 3 monomers in the asymmetric unit (Figure 5A) and protein interface surface analysis performed using the PISA server (https://www.ebi.ac.uk/msd-srv/prot_int/cgi-bin/piserver) showed that all protein-protein interfaces of the crystal lattice would not be stable in solution, confirming that YfiN_GGDEF_ is monomeric (as previously demonstrated in solution [18]). A magnesium ion is bound in the GTP binding site, coordinated by the oxygen atoms of D287, S288(CO), D330 (belonging to the GGDEF signature), and two water molecules (Figure 5B). Superposition with the recently solved structure of RmcA in complex with GTP and calcium [5], shows that the metal ions occupy the same position and that the two water molecules replace the oxygen provided by the β and γ phosphate groups of GTP (Figure 5C). The GTP substrate docks to the GGDEF domain in an extended conformation and a side reaction leading to the formation of c-GMP can be excluded given the position of the 3′-OH, which is too far from the α-phosphate to perform a nucleophilic attack. A closer look at the position of the phosphate groups with respect to the substrate-binding pocket indicates that the α-phosphate is particularly exposed to the solvent and, if the catalytically competent dimer does not form, may undergo a nucleophilic attack by a hydroxyl ion, thus producing GMP (Figure 5C).

## 4. Discussion

**Figure 6:**
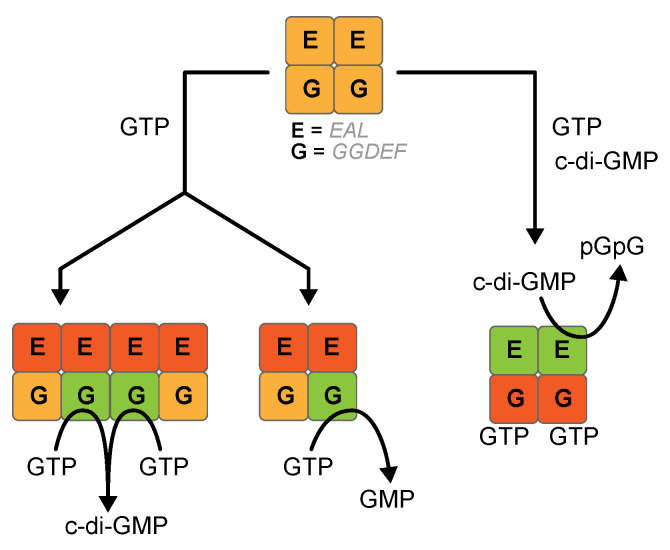
Scheme of the reactivity of DUAL RmcA with GTP, with or without c-di-GMP. E, EAL domain, G, GGDEF domain. In green the domains performing turnover, in red the domain in a resting state, in orange non-activated domain. It is not excluded that the red and the orange state of the EAL domain could show the same conformation.

Proteins involved in controlling c-di-GMP levels (and c-di-GMP itself) are characterized by an extraordinary plasticity and their function is deeply governed by allosteric control of other signals or domains. The structural analysis, where possible, reports on multiple conformation and oligomeric state governing protein function [3,29]. This feature makes the rigorous biochemical characterization of these proteins difficult, particularly from a quantitative point of view, slowing down our understanding of such potential therapeutic targets. To further complicate the scenario, the results reported in this study indicate that the GGDEF domain can exhibit a novel and unusual GTPase activity, related to its conformational frustration (see for a definition [30,31]). We observed this side-reaction both on GGDEF-EAL hybrid constructs and on a stand-alone GGDEF domain. In the first case, GTP-dependent GMP production is negatively affected by c-di-GMP, which in turn sustains the pGpG production. Thus, the apparent GTP → GMP conversion is likely to be an alternative to the PDE activity (see Figure 6 for a scheme). It is not the first time that a crosstalk between the EAL and the GGDEF domain takes place: according to mutagenesis studies on RmcA and structure data on both RmcA and RbdA, binding of GTP to GGDEF leads to a dramatic re-arrangement on the EAL domain, which, in turn, fasten the overall structure of the GGDEF-EAL tandem once c-di-GMP is bound to the EAL active site [5,8]. Once c-di-GMP levels drops (close to the K_M_ value measured for the EAL domain, [5]), the EAL active dimer is not fully saturated and likely dissociates (yielding an OFF conformation for the EAL); under these conditions, GTP-bound GGDEF is likely less conformationally constrained and may hydrolyse, as a monomeric entity, GTP into GMP. Structural data [5] suggest that under these conditions the GGDEF is constrained from interfacing with its other unit to produce c-di-GMP as for a DGC enzyme. The same fate probably is observed in the inactive PleD: if the GTP-bound active site is conformationally trapped as an isolated moiety, the (α-β)-GTPase activity takes place.

It is worth mentioning that the GGDEF reactivity deeply changes depending on the experimental setup: in the case of MorA, the experimental conditions reported in Phippen and coworkers includes a very high enzyme concentration (40 µM) and little GTP excess (100 µM), which promotes a DGC turnover. It is known that DGC reaction is dependent on protein concentration for both the active dimer formation step [18,28] and the cooperative substrate binding step [32]. The high protein concentration used for MorA, favoring stochastic intermolecular interactions between GGDEF domains, may randomly populate the active dimer for the c-di-GMP production, thus leading to an apparent DGC activity. Accordingly, the DGC phenotype is lost when using lower protein concentration, favoring the GTPase reaction. On the other hand, the results on RbdA raises some concerns on the characterization of the truncated GGDEF-EAL proteins and, possibly, on the GGDEF-containing proteins; the upstream domains are likely strategic to allow the GGDEF portion to enter DGC catalysis, as previously observed for the proteins containing the sole GGDEF, such as YfiN [18]. For YfiN, the conserved active site, confirmed by structural data, and the high affinity for the substrate are not sufficient to promote DGC, given that the conformational activation is hampered by the upstream domain(s) truncation.

Taken together, this analysis confirms that GGDEF-containing multi-domain proteins show not only conformational plasticity but also versatility in their activities at least in vitro: they may act as DGCs, GTP-dependent conformational switches or, to a lower extent, as GTPases (see Table S2 for k_cat_ comparison). The role of additional domains flanking the catalytic one is not new in the field of GTP-dependent proteins. In circularly permutated GTPases (cpGTPases) the presence on an additional domain located at the C-terminus with respect to the catalytic one is required [33] to prevent that an otherwise unfastened catalytic domain fails to stabilize GTP [34,35].

Up to now, we have no evidence that the GTPase activity has physiological relevance; most likely it is a spurious readout due to a certain experimental set up. According to in vitro parameters, GTPase occurs when c-di-GMP levels are low (close to the K_M_ of RmcA for c-di-GMP) [5]. Looking at the intracellular c-di-GMP levels and GTP cellular concentration even under starvation [9,36,37], the DUAL domain of RmcA could be predominantly in the GTP-bound state (considering the sub-µM affinity for GTP). Therefore, the eventual GTPase activity in the cellular background should be modulated mainly by a drop in the c-di-GMP levels; if relevant, its role may just prevent c-di-GMP consumption under a threshold of intracellular (or local) c-di-GMP concentration. At this stage, any further consideration on this aspect is over speculative and out of the scope of this work. Regardless of the physiological relevance of such activity, our experiments clearly indicate that characterization of the reactivity of GGDEF-containing proteins requires special caution, particularly in the protein engineering design stage.

## 5. Conclusions

Despite that the GGDEF domain is evolutionarily conserved and structurally stable, its function is strictly dependent on the neighboring domain(s) or sequences that, independently of their nature, are required to ensure the proper dimerization to enter DGC catalysis. It is possible to obtain recombinant stable and properly folded GGDEF domain from different proteins (and obtain crystals and solve the 3-D structure, as reported above), both in isolation or in combination with other domains, but their intrinsic reactivity is difficult to be clearly assessed. Their putative DGC and/or allosteric switch roles could be significantly affected or even abolished by the boundaries chosen in the engineering design step. Last but not least, the dependence on protein concentration should be carefully evaluated to characterize these proteins biochemically and to assign to a certain GGDEF domain its role in a multidomain protein.

## Figures and Tables

**Table 1:** Data collection and refinement statistics.

Data Collection	PA1120-E232start ^a^
Beamline	Bessy ID14.1
Space group	P2_1_2_1_2_1_
Cell dimension; a b c (Å), α β γ (°)	79.64 85.10 126.97 90.00 90.00 90.00
Resolution range (Å)	49.64–2.80 (2.95–2.80)
Half-set correlation CC(½) (%) ^b^	99.6 (68.2)
<I/σI>	10.5 (1.6)
Completeness (%)	100 (100)
N. unique reflection (total)	21,951 (3175)
Multiplicity	13.2 (13.5)
Wilson B-factor (Å^2^)	56.1
**Refinement**	
Resolution range (Å)	49.0–2.80
N° of unique reflections	19,705
R_free_ test set	2195 reflections (10.2%)
R_work_/R_free_ (%)	21.96/22.36
F_o_,F_c_ correlation	0.94
Total N. of atoms	3809
Average B-factor all atoms (Å^2^)	64.3
Bonds RMSD	
*Length (Å)*	0.007
*Angle (°)*	1.520
Ramachandran plot (n. residues, %)	
*Favoured*	485 (94.4)
*Allowed*	29 (5.6)

^a^ Values in parentheses refer to the highest-resolution shell; ^b^ Percentage of correlation between intensities from random half-data sets.

## Data Availability

The data presented in this study are available in the figures, in supplementary material and PDB databse.

## Supplementary Materials

The following are available online at https://www.mdpi.com/2075-1729/11/1/31/s1, Supplemental Methods; Figure S1: Identification of the product of GTP consumption by DUAL; Figure S2: Nucleotides content of MorA reaction(s).
