# Supplementary material for: Studying GGDEF Domain in the Act: Minimize Conformational Frustration to Prevent Artefacts
Source: Life (Basel). 2021 Jan 6;11(1):31. doi: 10.3390/life11010031 (PMC7825114; doi:10.3390/life11010031)
Supplement: Supplementary file 1 [file life-11-00031-s001.pdf]

SUPPLEMENTARY MATERIALS.

Supplemental methods.

HPLC-Mass measurements

HPLC-DAD-ESI-TRAP studies were performed using an Agilent 1100 series and an Ion Trap LC/MSD Trap SL G2445D from Agilent Technologies (Santa Clara, CA, USA) equipped with an ESI source operating in negative ionization mode. The separation of analytes was achieved on a C8 analytical column (250 × 4,6 i.d., 5 µm) from Agilent (USA). The mobile phase for HPLC-MS analyses was a mixture of water (A) and methanol (B) both containing 0.1% formic acid at 0.7 ml/min with an isocratic elution: 98% A : 2% B (total run time of 20 minutes). The injection volume was 5 µl performed with auto-sampler. The temperature of the column was 35 ℃ and diode array detection (DAD) was set on three wavelengths: 250, 240 and 230 nm. The temperature of the drying gas in the ionization source was 325 ℃, the gas flow was 12 l/min, the nebulizer pressure was 70 psi and the capillary voltage was 4000 V. Detection was performed in the SCAN mode from 50 to 1300 uma.

Table SI. Details of purification protocol optimized for each construct used in the present work.

Name	Lysis Buffer	Buffer A	FPLC Column	ε $(mg \cdot ml^{-1})$
LOV-DUAL	50 mM NaCl, 50 mM Tris-HCl pH 8, 1 mM PMSF and 1 cOmplete protease inhibitor (Roche)	20 mM Tris-HCl pH 7.5, 300 mM NaCl	Superdex 200	0.97
MorA; RbdA	250 mM NaCl, 50 mM Tris-HCl pH 8, 1mM PMSF, DNAsi 0.05 g1 and 1 cOmplete protease inhibitor (Roche)	150 mM NaCl, 20 mM Tris-HCl pH 7.6	Superdex 75	0.75; 0.68
PleD	20 mM Tris–HCl pH 8.0, 50 mM NaCl, 1 mM PMSF and 1 cOmplete protease inhibitor (Roche)	100 mM NaCl, 20 mM Tris–HCl pH 8.0	Superdex 200	0.3
$YfiN_{GGDEF}$	250 mM NaCl, 10 mM Tris-HCl pH 8, 10% v/v Glycerol, 2 mM PMSF and 1 cOmplete protease inhibitor (Roche)	250 mM NaCl, 10 mM Tris-HCl pH 8, 10% v/v Glycerol	Superdex 200	0.3

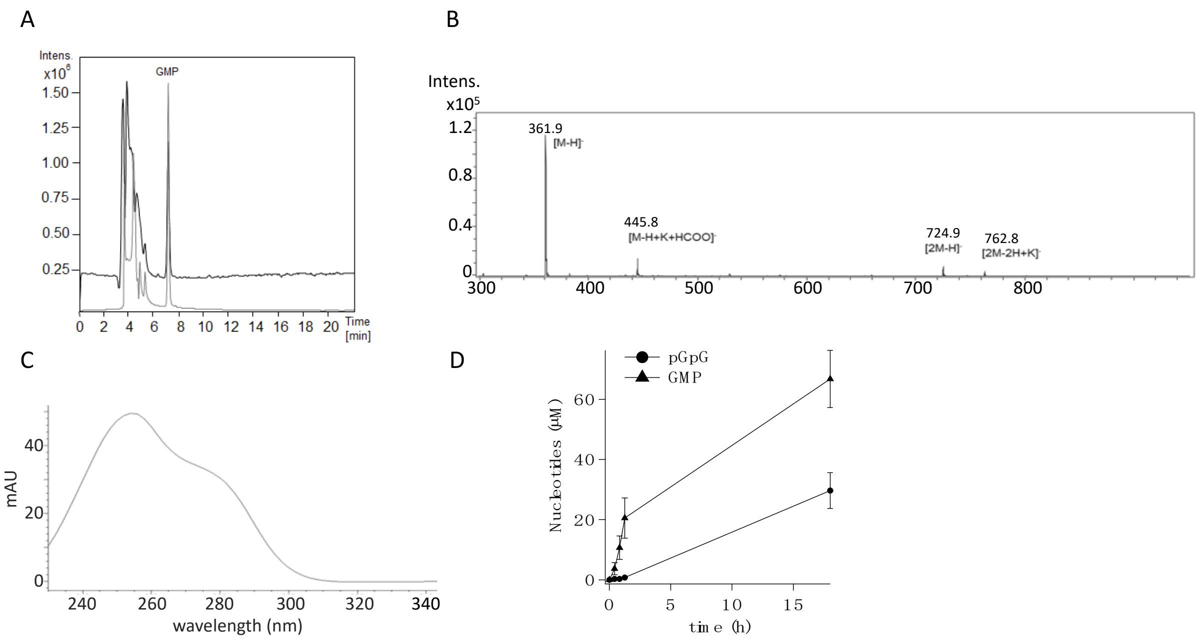

Figure S1. Identification of the product of GTP consumption by DUAL. (**A**) HPLC-MS chromatogram (black) and HPLC-DAD chromatogram (grey) monitored at 250 nm wavelength. The nucleotide content of the reaction mixture, containing 10 μM DUAL incubated with excess of GTP (100 μM) was separated by a C8 RP-HPLC, under the specific running conditions reported in Supplemental Materials and Methods. (**B**) Mass spectrum of GMP (peak retention time 7.034 min) recorded with ESI-Ion Trap and (**C**) UV spectrum from DAD of GMP (peak retention time 7.034 min). (**D**) The amount of the nucleotides observed in each chromatogram reported in Figure 1A has been plotted as a function of time (circles pGpG, triangles GMP). Data are the means of a triplicate ±SD.

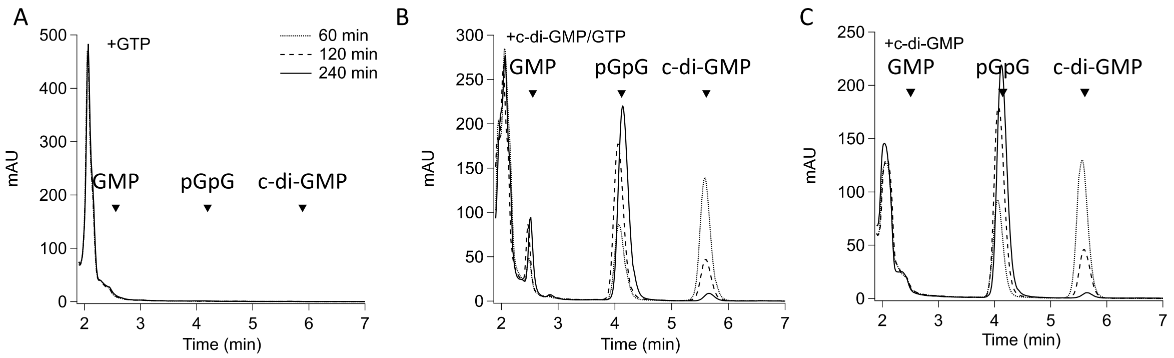

Figure S2. Nucleotides content of MorA reaction(s). HPLC-RP chromatograms of MorA diguanylate cyclase and phosphodiesterase activities carried out following the protocols of Phippen and coworkers [1]. Briefly, 40 µM of enzyme was incubated at room temperature in 50 mM Hepes pH 7.6, 300 mM NaCl, 2 mM MgCl2, 2 mM β-mercaptoethanol: to test the DGC activity (**A**), the enzyme was incubated with 100 µM GTP while it was incubated with 30 µM C-di-GMP to assay the PDE activity (**B**). The reaction was also performed incubating the enzyme with both nucleotides (**C**).

Table SII. Turnover number of selected DGCs. Observed GTPase k_{cat} obtained in this study are also reported in red for comparison, extrapolated from the observed activity at 1 h of reaction (in red).

Protein	Hybrid protein	K_{cat} (min^{-1})	References
PleD<br>*C. crescentus*	-	0.054	[2]
YddV (DosC)<br>*E. coli*	-	0.066	[3]
XAC0610<br>*Xanthomonas citri*	-	72	[4]
tDGC R158A*	-	2.6	[5]
RbdA<br>*P. aeruginosa*	✓	26<br>n.d.*	[6]
MorA<br>*P. aeruginosa*	✓	n.d.<br>0,05±0,01	
RmcA<br>*P. aeruginosa*	✓	-<br>0,035±0,01	

* mutant lacking product inhibition

* not detectable with the sole GTP

SUPPLEMENTAL REFERENCES.

1. Phippen, C.W.; Mikolajek, H.; Schlaefli, H.G.; Keevil, C.W.; Webb, J.S.; Tews, I. Formation and Dimerization of the Phosphodiesterase Active Site of the Pseudomonas Aeruginosa MorA, a Bi-Functional c-Di-GMP Regulator. *FEBS Letters* **2014**, doi:10.1016/j.febslet.2014.11.002.

2. Wassmann, P.; Chan, C.; Paul, R.; Beck, A.; Heerklotz, H.; Jenal, U.; Schirmer, T. Structure of BeF3--Modified Response Regulator PleD: Implications for Diguanylate Cyclase Activation, Catalysis, and Feedback Inhibition. *Structure* **2007**, *15*, doi:10.1016/j.str.2007.06.016.

3. Kitanishi, K.; Kobayashi, K.; Kawamura, Y.; Ishigami, I.; Ogura, T.; Nakajima, K.; Igarashi, J.; Tanaka, A.; Shimizu, T. Important Roles of Tyr43 at the Putative Heme Distal Side in the Oxygen Recognition and Stability of the Fe(II)-O2 Complex of YddV, a Globin-Coupled Heme-Based Oxygen Sensor Diguanylate Cyclase. *Biochemistry* **2010**, *49*, doi:10.1021/bi100733q.

4. Oliveira, M.C.; Teixeira, R.D.; Andrade, M.O.; Pinheiro, G.M.S.; Ramos, C.H.I.; Farah, C.S. Cooperative Substrate Binding by a Diguanylate Cyclase. *Journal of Molecular Biology* **2015**, *427*, 415–432, doi:10.1016/j.jmb.2014.11.012.

5. Rao, F.; Pasunooti, S.; Ng, Y.; Zhuo, W.; Lim, L.; Liu, A.W.; Liang, Z.X. Enzymatic Synthesis of C-Di-GMP Using a Thermophilic Diguanylate Cyclase. *Analytical Biochemistry* **2009**, *389*, doi:10.1016/j.ab.2009.03.031.

6. Liu, C.; Liew, C.W.; Wong, Y.H.; Tan, S.T.; Poh, W.H.; Manimekalai, M.S.S.; Rajan, S.; Xin, L.; Liang, Z.X.; Grüber, G.; et al. Insights into Biofilm Dispersal Regulation from the Crystal Structure of the PAS-GGDEF-EAL Region of RbdA from Pseudomonas Aeruginosa. *Journal of Bacteriology* **2018**, *200*, doi:10.1128/JB.00515-17.
